# Natural Selection in Diseases

**Published:** 1880-12

**Authors:** H. D. Valin

**Affiliations:** Chicago


### ARTICLE III.

### NATURAL SELECTION IN DISEASES. By H. D. VALIN, M.D., Chicago.

The conviction and common agreement of natural philosophers and eminent physicians are the evidences on which medical teachings are grounded; but neither law nor revelation should be considered as authorities in medicine.

Two entities are generally recognized in nature, viz., energy and inert matter; these two are called by some animated matter. The course of nature is the movement of inert matter when acted on by force; in other words, the regular progress of natural events. A natural cause, uniformly recurring, and producing similar results each time, on account of the accessory circumstances remaining the same, establishes a natural law; hence in the course of nature there are an indefinite number of such laws.

Natural selection, or the survival of the fittest, means that the organized being best qualified to escape, in a given medium, harmful circumstances, and destruction by the other species, survives in the struggle for life. But it does not mean that the survivor is the best in size, strength, beauty or virtue. This could be simplified by saying that a greater energy or force coming in conflict with a weaker one destroys it; and rats and bed-bugs are instances of some of the fittest animals who survived and multiplied to the greatest extent.

It could not be otherwise in the present constitution of the universe, except through the interference of a superior being; but, for practical purposes in medicine, supernatural interference is generally overlooked in its bearings on life and death. Indeed, all statistics in civilized countries refer diseases to natural causes without exception.

The author of the "Origin of Species," with a majority of later writers on the subject, does not recognize any premeditated results in the events of the world, and claims that the cause being brought to act, the result cannot be a deliberate one, but is necessary.

Human selection is deliberate to a certain extent, and consists in breeding from the choicest domestic animals, and the comparative improvement thus gained, as our perfected breeds of race-horses, merino sheep, etc. But the human species is very little influenced by natural selection as regards their struggle for life with other animals, on account of the great superiority of man over the latter, yet the struggle for life between nations is very great, and a frequent cause of war. Man is less influenced by human selection, because human charity takes a special care of weak individuals, which procreate as the rest. Lycurgus in Lacedemonia is said to have applied this law, through sacrificing the life of many individuals, with a good result. But the present emperor of Germany is said to have failed in his endeavor at raising a regiment of tall men of the age of his son.

Natural selection can be applied in some degree to all organisms, and, consequently, to the bacteria which are supposed to represent each infectious disease. But the spread of these cannot improve mankind; those most exposed, not necessarily the weakest, are mostly attacked. The wider these organisms spread, the greater the destruction of life; and finally, a majority of those attacked recover, often retaining an impaired vitality.

Immunity from infectious organisms is seldom met with. The Algerian sheep, it is true, enjoy such an immunity from splenic fever; and among mankind, four or five individuals, in every hundred, are said to resist the inoculation of small-pox, if they have not been vaccinated. An immunity from a second attack of some of those organisms, is well established, but has not been proven to be imparted to the progeny through inheritance.

A tendency to some diseases is transmitted by hereditary descent, because the progeny is modeled after the parents, and a lack of resistance to certain injurious causes will often be found in both, obviously. The conditions remaining the same, an increase in those diseases must take place; hence the well-known fact that two phthisical parents generally breed phthisical children; hence also the necessity for crossing, and the evil which sometimes results from the intermarriage of relatives. As an instance, a country settled by the descendants of various nations generally possesses a good breed, as the American people; and a secluded race declines, as the Negro or the Malay.

This recalls to one's mind that, although the course of nature is regular, it is far from being what mankind would have it. If man presided over the course of nature, it seems there would never have been a famine destroying so many millions of human beings as that of the Northern provinces of China in late years. Yet, it seems also that, without a relative evil, no good could exist.

Mankind being endowed with such wonderful qualities, take advantage of what good nature affords them, and have recourse to their genius and experience to guard against its evils. Hence architecture, garments, the culinary art, etc. Perhaps medicine could be referred to the same cause. We build houses to protect us from rain and snow, and fire enables us to live comfortably in cold climates. We also grind our corn before eating it, notwithstanding that we may contemplate the loss of our descendants' teeth in a remote futurity on that account.

In conclusion, breeding from weak or diseased individuals impairs the race. A frequent recurrence of epidemic diseases destroys the species. The improvement of mankind must be referred mostly to their morality, i. e., the accordance between the acts of a person and his notions of right. By this the individual has a tendency to progress, choosing the best of two given conditions. Hence the most intelligent and most righteous peoples are the most advanced in civilization, and have the best chances to survive. But what are the chances that the American Indians will survive in their conflict with civilized nations ?

An absolute protection against infectious diseases would greatly diminish the death rate of any country, and increase the average length of life. The immunity from a second or a third attack of some infectious diseases is a subject well worthy of the investigation of some able experimenter. It is also a useless truth that, destroying early all the weak and diseased individuals, would improve the race to a certain extent; but the existence of such individuals is often the cause of some good, being objects of charity, sympathy and generosity on the part of their more gifted relatives.

Finally, the best treatment of disease sometimes prolongs the latter, as preserving crippled individuals, and thereby augments the ratio of disease in some circumstances. But what evil is comparable to death for an intelligent being?

Many medical lecturers, pupils of the dogmatism of by-gone years, always see in the course of nature causes established in order to arrive at premeditated effects. If there is deliberation in the course of nature, sustaining such propositions, it would greatly benfit the modern physicians to get good proofs of it.

